# PSO–BiLSTM–Attention: An Interpretable Deep Learning Model Optimized by Particle Swarm Optimization for Accurate Ischemic Heart Disease Incidence Forecasting

**DOI:** 10.3390/bioengineering12121343

**Published:** 2025-12-09

**Authors:** Ruihang Zhang, Shiyao Wang, Wei Sun, Yanming Huo

**Affiliations:** 1 Graduate School, China Academy of Chinese Medical Sciences, Beijing 100102, China; ruihangzhang@cacmswj-edu.cn; 2 Department of Agrotechnology and Food Sciences, Wageningen University and Research, 6708 PB Wageningen, The Netherlands; evarze@126.com

**Keywords:** ischemic heart disease, incidence prediction, particle swarm optimization, Bidirectional Long Short-Term Memory networks, attention mechanism, deep learning, interpretability

## Abstract

Ischemic heart disease (IHD) remains the predominant cause of global mortality, necessitating accurate incidence forecasting for effective prevention strategies. Existing statistical models inadequately capture nonlinear epidemiological patterns, while deep learning approaches lack clinical interpretability. We constructed an interpretable predictive framework combining particle swarm optimization (PSO), bidirectional long short-term memory (BiLSTM) networks, and a novel multi-scale attention mechanism. Age-standardized incidence rates (ASIRs) from the Global Burden of Disease (GBD) 2021 database (1990–2021) were stratified across 24 sex-age subgroups and processed through 10-year sliding windows with advanced feature engineering. SHapley Additive exPlanations (SHAP) provided a three-level interpretability analysis (global, local, and component). The framework achieved superior performance metrics: mean absolute error (MAE) of 0.0164, root mean squared error (RMSE) of 0.0206, and R^2^ of 0.97, demonstrating a 93.96% MAE reduction compared to ARIMA models and a 75.99% improvement over CNN–BiLSTM architectures. SHAP analysis identified females aged 60–64 years and males aged 85–89 years as primary predictive contributors. Architectural analysis revealed the residual connection captured 71.0% of the predictive contribution (main trends), while the BiLSTM–Attention pathway captured 29.0% (complex nonlinear patterns). This interpretable framework transforms opaque algorithms into transparent systems, providing precise epidemiological evidence for public health policy, resource allocation, and targeted intervention strategies for high-risk populations.

## 1. Introduction

IHD remains one of the most critical public health issues globally. It is a cardiac condition caused by insufficient coronary artery blood supply, characterized by severe pathology, complex complications, and poor prognosis, representing one of the most common types of cardiovascular diseases (CVD) [1,2]. In 2023, the global number of CVD patients reached 626 million, of which approximately 239 million were IHD patients [3]. According to World Health Organization statistics, IHD causes approximately 9 million deaths annually, accounting for over 16% of total global mortality [4,5,6]. With the acceleration of population aging, the spread of unhealthy lifestyles, and the epidemic of metabolic diseases, the disease burden of IHD demonstrates a continuously rising trend, posing severe challenges to global public health systems [7,8]. IHD not only seriously impacts patients’ quality of life but also imposes heavy economic burdens on families and society [9]. Accurate prediction of IHD incidence trends holds significant strategic importance and practical value for formulating forward-looking prevention and control strategies, optimizing healthcare resource allocation, and evaluating the effectiveness of intervention measures [10,11,12].

### 1.1. Review of Related Work

Traditional epidemiological prediction methods mainly rely on statistical models, such as ARIMA and the gray model (GM). These methods demonstrate stable performance in capturing linear trends but exhibit obvious limitations when confronted with complex nonlinear time series patterns [13]. ARIMA and its variants model stationary or differenced stationary data by analyzing the autocorrelation structure of time series [14,15]. Wang et al. [16] applied ARIMA to predict tuberculosis incidence with satisfactory short-term results. However, traditional methods predominantly employ univariate modeling and cannot effectively integrate the interactive effects of multi-dimensional features [17,18].

Machine learning methods provide more flexible modeling frameworks for disease prediction. Support vector regression (SVR) demonstrates excellent performance in small-sample scenarios, with chronic heart failure research showing that ACP parameters predicted mortality with an AUC of 0.770 [19]. Random forest models predicted acute myocardial infarction with an accuracy of 0.97 and AUC of 0.96 [20]. XGBoost demonstrated powerful capabilities with a risk ratio of 1.18 [21]. In addition to prediction, these methods are widely applied in diagnosis, where comprehensive surveys have highlighted various algorithms for coronary artery disease detection [22], and comparative studies have shown the high efficiency of models like random forest and SVM in that diagnostic capacity [23]. However, these “static” learning paradigms still exhibit deficiencies in time series modeling, which is the focus of this paper.

Deep learning has achieved breakthrough progress in time series prediction by constructing multi-layer nonlinear transformation networks [24,25]. A recurrent neural network (RNN) captures sequence dependencies but is constrained by vanishing gradient problems [26,27]. LSTM networks solved this problem through gating mechanisms, with pulse wave analysis achieving 0.8595 accuracy by employing the SHAP framework [28]. BiLSTM further enhances performance through bidirectional information encoding, predicting heart failure events with an AUC of 0.977 [29]. Attention mechanisms enable adaptive focus on critical sequence segments, reducing MAPE to 15.15% [30].

Although deep learning has achieved significant breakthroughs, hyperparameter selection remains a critical bottleneck. PSO has been widely adopted due to its simplicity, efficiency, and strong global search capability [31]. Xu et al. [32] employed PSO to optimize LSTM models for lung cancer incidence prediction, significantly improving accuracy. However, the “black box” nature of deep learning limits its application in medical decision-making [33]. The SHAP framework, based on game-theoretic Shapley values, provides a theoretically guaranteed fair attribution method [34]. Recent studies indicate that SHAP demonstrates powerful explanatory capabilities in drug development and disease prediction [34,35]. Specifically in cardiovascular research, several studies have begun to apply SHAP and other interpretability methods for risk prediction using clinical and dietary data [36] or feature decomposition [37]. These studies pave the way for interpretable models that can ‘open the black box’, which is a key objective our current work builds upon in the specific domain of forecasting epidemiological trends.

Existing research still exhibits deficiencies: insufficient consideration of population heterogeneity [38], limited model architecture innovation, hyperparameter optimization reliance on manual experience, and insufficient model interpretability.

### 1.2. Main Contributions and Innovations

This study aims to address the challenges of insufficient nonlinear processing capability and the “black box” problem faced by traditional models in IHD incidence forecasting. The core contributions and innovations of this study are concentrated in the following four areas:

(1) Model Architecture Innovation: We designed a deep learning architecture integrating BiLSTM and a novel multi-scale attention mechanism. This architecture can simultaneously capture long-term dependencies in disease development and complex patterns at different time scales (short-, medium-, and long-term), and handles the heterogeneity of 24 sex-age groups through a group-specific layer.
(2) Intelligent Optimization Strategy: We systematically applied the PSO algorithm to the complex IHD incidence prediction model. Through automated hyperparameter tuning for the BiLSTM–Attention network, it overcomes the subjectivity of traditional manual tuning, improves search efficiency by over 70%, and enhances the model R^2^ from 0.93 to 0.97.
(3) Systematic Interpretability Innovation: Building on existing work in interpretable ML for CVD prediction [36,37], we constructed a systematic three-level “global–local–component” SHAP interpretability framework. This framework provides theoretically fair attribution based on game-theoretic Shapley values, not only revealing key predictive features (e.g., “females aged 60–64”) but also quantifying the contributions of internal model components, transforming the “black box” model into a transparent clinical decision-support tool.
(4) Application Scenario Innovation: We constructed a high-precision prediction model covering a 32-year long-term series and 24 finely stratified sex-age groups. This provides precise epidemiological evidence for formulating differentiated prevention and control strategies for different populations.

## 2. Data and Methods

### 2.1. Data Source and Preprocessing

The data for this study were sourced from the GBD 2021 database. The GBD project, led by the Institute for Health Metrics and Evaluation at the University of Washington, represents the most authoritative and comprehensive disease burden assessment system globally, encompassing systematic epidemiological data from 204 countries and territories, covering 369 diseases and injuries, and 87 risk factors [39]. This study obtained the Age-Standardized Incidence Rate (ASIR) data for IHD in China from 1990 to 2021 through the GHDx platform (https://ghdx.healthdata.org/, accessed on 7 September 2025). ASIR undergoes age standardization using the world standard population, eliminating the influence of population age structure differences across different periods. Data units are expressed as the number of incident cases per 100,000 population, providing point estimates and 95% uncertainty intervals. Data were stratified by sex (male/female) and 12 GBD age groups (40–44 to ≥95 years) (focusing on ≥40 years due to low IHD incidence and high data fluctuation under 40), forming 24 time series (32 annual observations each). Preprocessing included five steps:

1. Cleaning/quality control: No missing values; outliers (boxplot: 3 × IQR) retained as genuine events; ADF tests showed non-stationary series.
2. Standardization: Z-score normalized data (mean = 0, SD = 1) to eliminate magnitude differences.
3. Feature engineering: Five derived feature types expanded the 24D to 144D. These were as follows:
  (1) Trend: Three-, five-, and seven-year moving averages to capture long-term tendencies.
  (2) Residual: Short-term fluctuations (original value minus three-year moving average).
  (3) Volatility: Three-year rolling standard deviation as a metric for uncertainty.
  (4) Sex Difference: This feature was constructed for each age group i by calculating (Male_ASIR_i—Female_ASIR_i). This value was appended to the feature vector for both the male and female groups of that age. This explicitly provides the model with the magnitude of the sex-based disparity, as the gap between sexes (whether it is widening or converging) is itself a critical predictive pattern.
  (5) Smoothing: Exponentially weighted moving average (EWMA, decay = 0.3) to provide a smoothed recent trend.
4. Modeling strategy: Ten-year sliding window (one-year step) converted data to supervised format.
5. Dataset splitting: A sequential split (8:2) was used, with 1990–2016 (27 years) for training and 2017–2021 (5 years) for the hold-out test set. This “walk-forward” method is standard for time series forecasting as it prevents information leakage. While rolling cross-validation is an alternative, it was deemed computationally infeasible given the high cost of re-running the full PSO for each fold. The 2017–2021 test period was intentionally chosen as it is the most recent data available, providing a robust test of the model’s ability to generalize to real-world, unseen data, including any fluctuations from recent global events like the COVID-19 pandemic (2020–2021).

**Figure 1:**
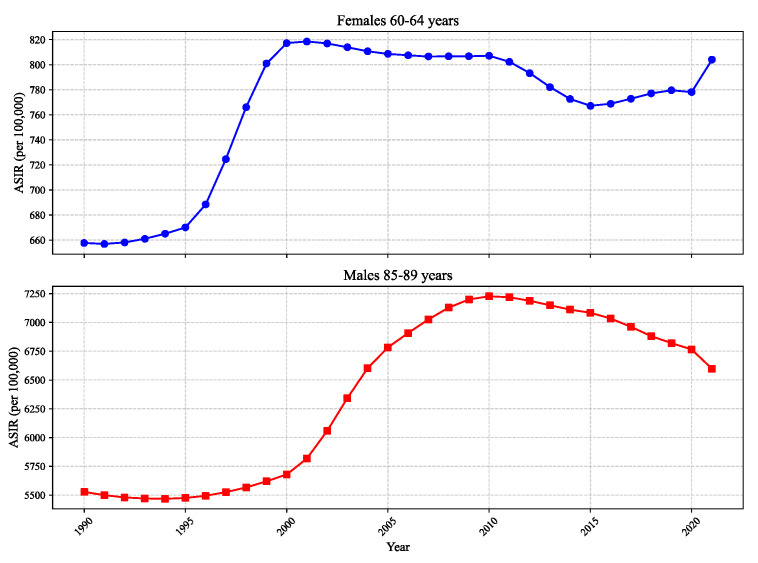
Example time series data for IHD age-standardized incidence rates (ASIRs) from 1990 to 2021 for two key demographic groups, illustrating different scales and trends.

To provide a clearer understanding of the data, Figure 1 plots representative time series samples from the dataset. The plots for “Females 60–64 years” and “Males 85–89 years” are shown, as these were identified by our model (see Section 3.3.1) as key predictive groups. The plots illustrate the distinct nonlinear trends and volatility patterns that the model must learn to capture.

### 2.2. Methods

**Figure 2:**
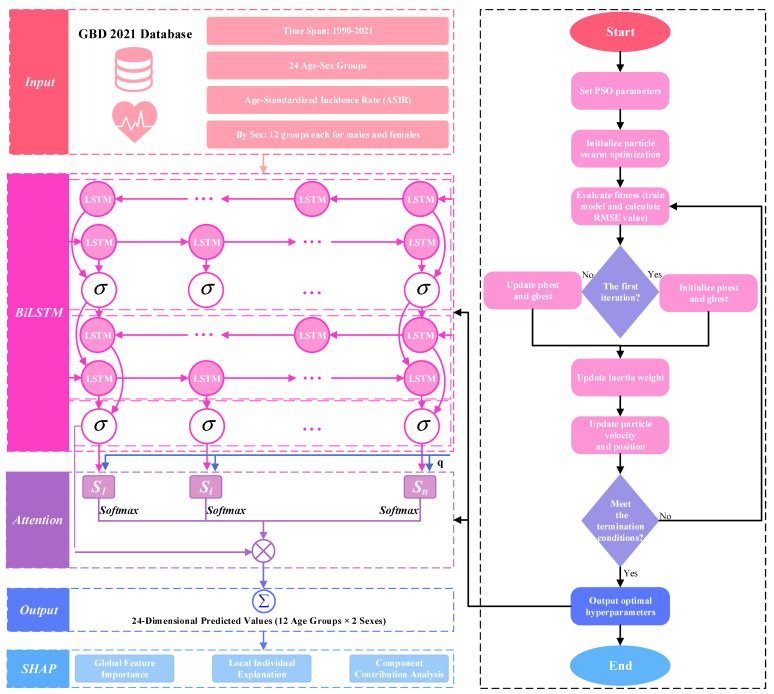
Technical roadmap of the research.

This study proposes a PSO–BiLSTM–Attention hybrid deep learning model for precise IHD incidence prediction, integrating temporal modeling, feature selection, and intelligent hyperparameter optimization into a complete framework of “data-driven + intelligent optimization + interpretability analysis.” Figure 2 illustrates the overall technical roadmap of the research, encompassing four core components: data input, model training, hyperparameter optimization, and interpretability analysis.

#### 2.2.1. Input Layer

The input layer receives preprocessed time series data as a three-dimensional tensor B × T × D, where B is batch size, T = 10 is sequence length, and D = 144 is feature dimensionality (24 original features plus 120 derived features including trends, residuals, volatility, sex differences, and smoothing features).

The input layer projects features into hidden space through the following:

(1) Hinput=ReLU(X⋅Winput+binput)

where $W_{input}\in {\mathbb{R}}^{144\times d_{h}}$ is the weight matrix, $b_{input}\in {\mathbb{R}}^{d_{h}}$ is the bias vector, and $d_{h}$ is the hidden layer dimensionality (set to 64 after PSO). ReLU is the activation function. Batch normalization accelerates convergence and improves stability.

#### 2.2.2. BiLSTM Module

LSTM is a recurrent neural network architecture specifically designed for processing time series data, effectively solving the vanishing gradient and long-term dependency problems of traditional RNN through the introduction of gating mechanisms. An LSTM unit contains three gating structures: forget gate, input gate, and output gate, as well as a cell state for storing long-term information. The forward propagation process of LSTM is described by the following mathematical formulas:

The forget gate decides which historical information needs to be discarded:

(2) $$f_{t}=\sigma (W_{f}\cdot [h_{t-1},x_{t}]+b_{f})$$

The input gate decides which new information needs to be stored:

(3) $$i_{t}=\sigma (W_{i}\cdot [h_{t-1},x_{t}]+b_{i})$$

(4) $${\tilde{C}}_{t}=\tanh(W_{C}\cdot [h_{t-1},x_{t}]+b_{C})$$

The memory cell update combines both forgetting and input information:

(5) $$C_{t}=f_{t}⊙C_{t-1}+i_{t}⊙{\tilde{C}}_{t}$$

The output gate controls the hidden state output at the current time step:

(6) $$o_{t}=\sigma (W_{o}\cdot [h_{t-1},x_{t}]+b_{o})$$

(7) $$h_{t}=o_{t}⊙\tanh(C_{t})$$

where $x_{t}$ is the input feature at time step t, $h_{t}$ is the hidden state output, $C_{t}$ is the cell state, σ is the Sigmoid activation function, ⊙ represents element-wise multiplication (Hadamard product), and W and b are the weight matrix and bias vector, respectively.

**Figure 3:**
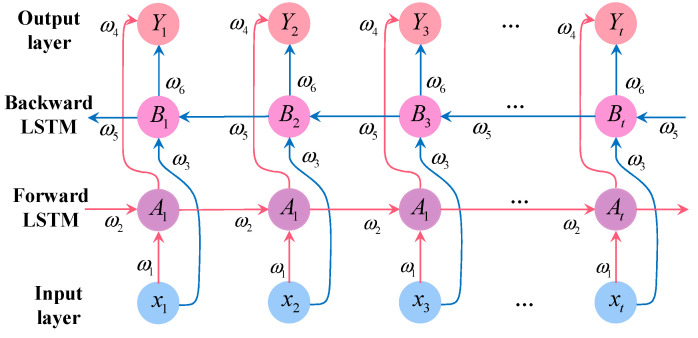
BiLSTM structure.

Standard LSTM captures only past-to-future dependencies. BiLSTM resolves this limitation by constructing two independent LSTM layers in forward and backward directions. As shown in Figure 3, the forward layer of BiLSTM processes the sequence in chronological order t=1→T, capturing the influence of historical information on the current state; the backward layer processes the sequence in reverse order t=T→1, capturing the influence of future information on the current state. The hidden states from both directions are fused through concatenation:

(8) $$\vec{h_{t}}=LSTM_{forward}(x_{t},\vec{h_{t-1}})$$

(9) $$\overset{\leftarrow }{h_{t}}=LSTM_{backward}(x_{t},\overset{\leftarrow }{h_{t+1}})$$

(10) $$h_{t}=[\vec{h_{t}};\overset{\leftarrow }{h_{t}}]$$

where [⋅;⋅] represents the vector concatenation operation. The concatenated hidden state $h_{t}\in {\mathbb{R}}^{2d_{h}}$ simultaneously contains temporal information from both forward and backward directions, forming a richer feature representation.

This study uses a stacked BiLSTM (two layers, optimized by PSO), with the first layer’s output as the second layer’s input to extract high-level features. A total of 15% dropout is used between layers to prevent overfitting.

#### 2.2.3. Attention Mechanism

**Figure 4:**
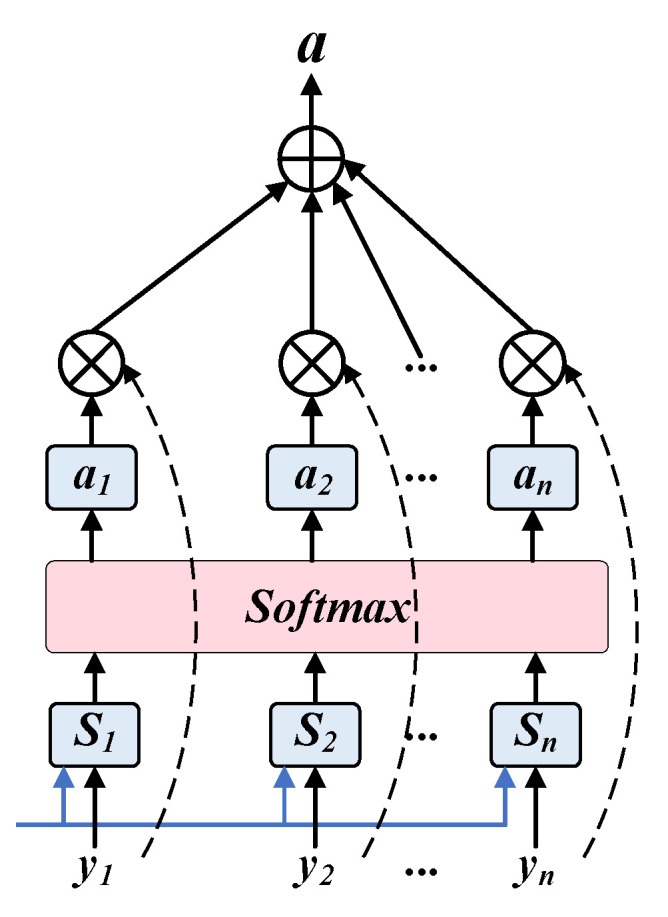
Attention structure.

The attention mechanism learns dynamic weights to focus on key time nodes in long sequences, improving prediction accuracy and interpretability. It must be emphasized that while the basic calculations of the attention mechanism (as shown in Formulas (11)–(13)) are common methods in deep learning, the contribution of this study lies in designing and implementing a novel multi-head multi-scale attention mechanism. Traditional attention mechanisms typically operate on a single scale, whereas the mechanism designed in this study includes three attention modules at different scales, each containing four attention heads. As shown in Figure 4, these modules are designed to focus, respectively, on capturing the temporal patterns of short-term fluctuations (1–3 years), medium-term trends (3–6 years), and long-term evolution (6–10 years) in the IHD incidence data.

First, the attention score for each time step is calculated through a fully connected layer:

(11) $$s_{t}=\tanh(W_{a}\cdot h_{t}+b_{a})$$

(12) $$a_{t}=soft\max(\frac{s_{t}\cdot v_{a}^{T}}{\sqrt{d_{k}}})$$

where $W_{a}\in {\mathbb{R}}^{2d_{h}\times d_{a}}$ is the attention weight matrix, $v_{a}\in {\mathbb{R}}^{d_{a}}$ is the learnable query vector, and $d_{k}$ is the scaling factor to prevent gradient vanishing. The Softmax function ensures that the sum of attention weights across all time steps equals one.

Subsequently, based on the attention weights, a weighted aggregation of BiLSTM hidden states is performed to generate the context vector:

(13) $$c=\sum_{t=1}^{T}a_{t}\cdot h_{t}$$

The context vector $c\in {\mathbb{R}}^{2d_{h}}$ is the weighted average of features from all time steps, with time steps having larger weights contributing more significantly to the final representation. To enhance the model’s capability to capture multi-temporal scale patterns, this study further extends to a multi-head multi-scale attention mechanism. Specifically, three attention modules at different scales are constructed, with each scale containing four attention heads, focusing, respectively, on short-term fluctuations (1–3 years), medium-term trends (3–6 years), and long-term evolution (6–10 years). The context vectors from each scale are fused through concatenation:

(14) $$C_{attention}=[c_{1};c_{2};c_{3}]$$

#### 2.2.4. PSO Module

The effectiveness of deep learning models relies heavily on hyperparameters. Traditional search methods are inefficient. This study uses PSO, a swarm intelligence-based algorithm inspired by bird foraging. PSO has N particles, each a hyperparameter solution, updating via individual and global best positions. Formulas (15)–(17) describe the standard update process of the PSO algorithm.

The contribution of this study is not in proposing these general optimization equations, but rather in innovatively applying the PSO algorithm to the complex BiLSTM–Attention hybrid model for systematic, automatic hyperparameter tuning. Faced with a multi-dimensional and highly complex hyperparameter space—including the number of BiLSTM layers, hidden units, dropout rate, learning rate, and attention heads—traditional manual tuning or grid search is inefficient and prone to local optima.

This study utilizes the global search capability of PSO, using the model’s validation set root mean squared error (RMSE) as the fitness function, to systematically and automatically search for the optimal hyperparameter combination. This customized optimization strategy is one of the key points distinguishing this research, ensuring that the PSO–BiLSTM–Attention model can achieve its optimal performance. The velocity and position update formulas for particle i at the *k*-th iteration are as follows:

(15) $$v_{i}^{k+1}=w\cdot v_{i}^{k}+c_{1}\cdot r_{1}\cdot (p_{best,i}-x_{i}^{k})+c_{2}\cdot r_{2}\cdot (g_{best}-x_{i}^{k})$$

(16) $$x_{i}^{k+1}=x_{i}^{k}+v_{i}^{k+1}$$

where w is the inertia weight, controlling the algorithm’s global exploration and local exploitation capabilities; $c_{1}$ and $c_{2}$ are learning factors, separately controlling the speed at which particles move toward the individual best and global best; r_1_ and r_2_ are random numbers in the interval [0, 1], introducing stochasticity to avoid premature convergence. This study employs an adaptive inertia weight strategy with linear decay over iterations:

(17) $$w(k)=w_{\max}-\frac{w_{\max}-w_{\min}}{K_{\max}}\cdot k$$

where $w_{\max}=0.9,\;w_{\min}=0.4,\;K_{\max}=20$ is the maximum number of iterations. This strategy ensures that the algorithm conducts broad exploration in the early stages and aggregates toward local refinement in the later stages.

The PSO process is as follows: (1) Initialize the positions and velocities of eight particles, randomly distributed in the hyperparameter search space. (2) Train the BiLSTM–Attention model corresponding to each particle and evaluate performance on the validation set (using RMSE as the fitness function). (3) Update the individual best and global best for each particle. (4) Adjust particle positions according to the velocity update formula. (5) Determine whether the maximum number of iterations or convergence conditions have been reached; if not, return to step (2). (6) Output the global optimal hyperparameter configuration.

#### 2.2.5. Output Layer

The output layer maps the high-dimensional feature vectors generated by the attention mechanism into final prediction results. Considering that this study needs to simultaneously predict incidence rates for 24 sex-age groups, the output layer adopts a group-specific layer design, constructing independent prediction pathways for each group to fully account for the heterogeneous characteristics of different populations.

Specifically, the output from the attention module first undergoes feature transformation through a fully connected layer:

(18) Hshared=ReLU(Cattention⋅Wshared+bshared)

Subsequently, 24 group-specific fully connected layers process the shared feature representation in parallel, generating predictions for each group:

(19) $${\hat{y}}_{g}=H_{shared}\cdot W_{g}+b_{g},\;\;g=1,2,\ldots ,24$$

where $W_{g}\in {\mathbb{R}}^{d_{shared}\times 1}$ is the dedicated weight vector for group g. This design enables the model to learn specific patterns for each age group while simultaneously achieving knowledge transfer through shared underlying features.

To further enhance prediction stability, this study introduces the residual connection mechanism. Residual connections directly project the linear projection of the input features onto the output of the deep network, constructing a dual-pathway prediction architecture:

(20) $$\hat{Y}=\alpha \cdot {\hat{Y}}_{BiLSTM-Attention}+(1-\alpha )\cdot (X_{avg}\cdot W_{residual})$$

where $X_{avg}$ is the average of the input sequence along the time dimension, $W_{residual}$ is the residual projection matrix, and α is a learnable fusion weight. The residual connection pathway directly captures the main trends in the data, while the BiLSTM–Attention pathway captures complex nonlinear patterns and local fluctuations, with the two complementing each other to achieve optimal prediction.

Finally, to enhance the diversity of ensemble learning, three sub-models with slight parameter perturbations are trained, with the final prediction obtained through simple average fusion:

(21) $${\hat{Y}}_{ensemble}=\frac{1}{3}\sum_{m=1}^{3}{\hat{Y}}_{m}$$

The ensemble strategy effectively reduces the overfitting risk of single models and improves the robustness of prediction results.

#### 2.2.6. SHAP Interpretability Analysis Framework

To enhance model transparency and credibility, this study introduces the SHAP method to conduct comprehensive interpretability analysis of the model. The mathematical definition of SHAP values is as follows:

(22) $$\phi_{j}=\sum_{S\subseteq F\backslash \{j\}}\frac{|S|!(|F|-|S|-1)!}{|F|!}[f(S\cup \{j\})-f(S)]$$

where *F* is the set of all features, *S* is the subset of features not including feature j, and μ is the model’s predicted value using only the feature subset *S*. This formula calculates the average marginal contribution of feature j across all possible feature combinations, ensuring fairness and consistency.

For deep neural network models, this study employs the GradientExplainer method to approximate SHAP values. This method rapidly estimates feature contributions based on the model’s gradient information and reference samples (background data):

(23) $$\phi_{j}\approx \frac{\left(x_{j}-x_{j}^{ref})\cdot \nabla_{x_{j}}f(x\right)}{M}\sum_{m=1}^{M}1$$

where $x_{j}^{ref}$ is the reference value for the j-th feature (typically the training set mean), and $\nabla_{x_{j}}f(x)$ is the gradient of the model output with respect to that feature.

## 3. Results and Discussion

This section details the experimental setup, model performance, and interpretability analysis. The primary outcome of this study is the development of the PSO–BiLSTM–Attention model, which achieved superior performance across all evaluation metrics (MAE: 0.0164, RMSE: 0.0206, R^2^: 0.97) on the 2017–2021 test set. As detailed in the ablation and comparative analyses, our model demonstrated significant improvements over both of its own components and existing state-of-the-art models, validating the effectiveness of its hybrid architecture and intelligent optimization.

### 3.1. Experimental Setup

#### 3.1.1. Evaluation Metrics

To comprehensively and objectively evaluate the prediction performance of the PSO–BiLSTM–Attention hybrid model proposed in this study, five commonly used regression evaluation metrics in the field of time series forecasting are employed.

It needs to be clarified here that formulas 24 to 28 (MAE, MSE, RMSE, MAPE, R^2^) are standard and general metrics for measuring the performance of regression models. This study uses these recognized metrics to objectively quantify, under a unified benchmark, the significant advantages in prediction accuracy and generalization capability of the model proposed in this study compared to traditional models like ARIMA and SVR, as well as other deep learning models like LSTM and CNN–BiLSTM (as shown in Tables 2 and 3). The comprehensive use of these metrics ensures the fairness and reliability of the model comparison.

1. MAE:

(24) $$MAE=\frac{1}{n}\sum_{i=1}^{n}\left|y_{i}-{\hat{y}}_{i}\right|$$

2. Mean squared error (MSE):

(25) $$MSE=\frac{1}{n}\sum_{i=1}^{n}{\left(y_{i}-{\hat{y}}_{i}\right)}^{2}$$

3. RMSE:

(26) $$RMSE=\sqrt{\frac{1}{n}\sum_{i=1}^{n}{\left(y_{i}-{\hat{y}}_{i}\right)}^{2}}$$

4. Mean absolute percentage error (MAPE):

(27) $$MAPE=\frac{100\%}{n}\sum_{i=1}^{n}\left|\frac{y_{i}-{\hat{y}}_{i}}{y_{i}}\right|$$

5. Coefficient of determination (R^2^):
  (28) $$R^{2}=1-\frac{\sum_{i=1}^{n}{\left(y_{i}-{\hat{y}}_{i}\right)}^{2}}{\sum_{i=1}^{n}{\left(y_{i}-\bar{y}\right)}^{2}}$$
  where n represents the number of samples, $y_{i}$ represents the true value of the *i*-th sample, ${\hat{y}}_{i}$ represents the predicted value of the *i*-th sample, and $\bar{y}$ represents the mean of all sample true values.

#### 3.1.2. Parameter Settings

Experiments were conducted on Windows 10 with Intel Xeon E5-2697 v4 processor, 64 GB RAM, NVIDIA RTX 4090 GPU, using the Python (3.12) and PyTorch (2.0.1) framework. The dataset was split 8:2 (training: testing) with a 10-year sliding window and 1-year prediction step. PSO algorithm optimized hyperparameters with linear decay inertia weight and learning factors of 2.0. Optimal parameters included the following: hidden dimension 64, two BiLSTM layers, 0.15 dropout ratio, and 0.002 learning rate. Input features expanded from 24 to 144 dimensions through feature engineering (trend, residual, volatility, sex-difference, and smoothing components). Multi-scale attention employed three scales with four heads each. Training used Adam optimizer with cosine annealing scheduling, early stopping, and gradient clipping. An ensemble of three sub-models improved stability through averaging. SHAP interpretability analysis used GradientExplainer with data augmentation.

#### 3.1.3. Parameter Justification and Reproducibility

To enhance clarity and reproducibility, Table 1 summarizes the justification for key methodological choices and hyperparameters discussed in Section 2.1 and Section 2.2.

#### 3.1.4. Computational Complexity Analysis

The computational complexity of the proposed model is primarily driven by three components: the BiLSTM layers, the Attention mechanism, and the PSO algorithm.

(1) BiLSTM–Attention (Training): The complexity for a single training step is dominated by the BiLSTM layers. This is proportional to the sequence length (10), the input feature dimension (144), and the square of the hidden dimension (64). The multi-head attention mechanism adds a secondary complexity, which is not dominant in this case as the sequence length is small.
(2) PSO (Optimization): The overall complexity of the optimization phase is determined by the number of PSO iterations (20), multiplied by the number of particles (8), and the cost of evaluating one particle (i.e., training and validating the model once). While this optimization phase is computationally expensive, it is an offline process. The final, trained model’s prediction (inference) time is very fast, suitable for real-world application.

### 3.2. Model Performance Analysis

#### 3.2.1. Ablation Experiments

Systematic ablation experiments validated each component’s effectiveness in the PSO–BiLSTM–Attention model. Five configurations were compared: LSTM, BiLSTM, BiLSTM–Attention, PSO–BiLSTM, and complete PSO–BiLSTM–Attention. Table 2 presents the performance of each model across five evaluation metrics.

A key observation in Table 2 is the exceptionally large performance improvement when moving from PSO–BiLSTM (MAE: 0.0678) to the full PSO–BiLSTM–Attention model (MAE: 0.0164). This massive jump is attributed to the limitations of a standard BiLSTM in handling long sequences. Without attention, the BiLSTM model suffers from an information bottleneck, as it must compress all information from the 10-year input sequence into its final hidden state. The addition of our multi-scale attention mechanism (Section 2.2.3) resolves this. It allows the model to “look back” and dynamically assign importance to specific time steps (e.g., short-term fluctuations, medium-term trends, and long-term evolution) from the 10-year history. This ability to access and weigh the most salient temporal patterns, rather than relying on a compressed summary, explains the dramatic reduction in error.

Table 2 shows progressive performance improvements with component additions. Basic LSTM achieved MAE of 0.1106 and R^2^ of 0.86. BiLSTM improved to MAE 0.0994 and R^2^ 0.89, validating bidirectional modeling. BiLSTM–Attention further reduced MAE to 0.0919 (R^2^ 0.92), demonstrating attention’s effectiveness in capturing key temporal features. PSO–BiLSTM achieved MAE 0.0678 and R^2^ 0.94 through intelligent hyperparameter optimization. The complete PSO–BiLSTM–Attention model achieved optimal performance: MAE 0.0164, MSE 0.0426, RMSE 0.0206, MAPE 15%, and R^2^ 0.97—representing 85.2% MAE reduction and 12.8% R^2^ improvement over basic LSTM.

**Figure 5:**
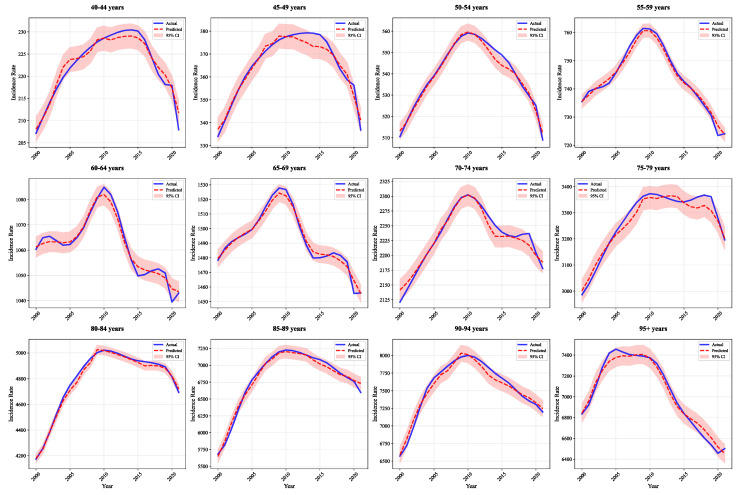
Actual and predicted IHD incidence rates in males by age.

**Figure 6:**
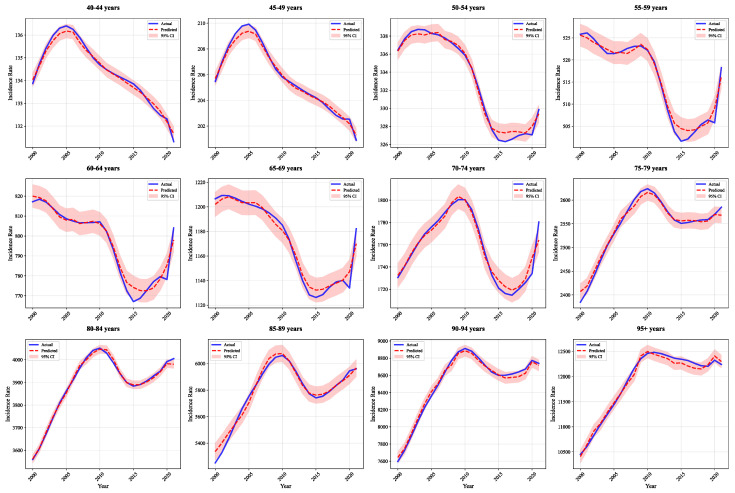
Actual and predicted IHD incidence rates in females by age.

Figure 5 and Figure 6 demonstrate high-precision fitting across all 12 male and female age groups, with prediction curves closely matching actual values and narrow 95% confidence intervals. The model accurately captures trends even in elderly groups (≥75 years) with large fluctuations, demonstrating strong generalization. This performance stems from group-specific layers that construct independent prediction pathways for each sex-age group, accounting for population heterogeneity.

#### 3.2.2. Comparative Analysis with Previous Studies

To comprehensively evaluate the advancement of the PSO–BiLSTM–Attention model, we conducted a systematic comparison against four representative models cited in previous studies: the classic statistical model ARIMA [40], the traditional machine learning model SVR [41], and two deep learning models, CNN–LSTM [42] and CNN–BiLSTM [43]. To address critical concerns regarding comparison fairness, we clarify the following methodological points that ensure all baseline comparisons are conducted under identical and optimal conditions:

(1) Dataset and Data Split: We did not use the performance metrics reported in [40,41,42,43], as those studies used different datasets, time periods, and population cohorts. Instead, we re-implemented all four baseline models from scratch and trained and tested them on our exact same GBD 2021 dataset. All models, including our own, used the identical 8:2 sequential training–testing split (1990–2016 for training, 2017–2021 for testing). This “walk-forward” methodology strictly prevents temporal information leakage and ensures all models are evaluated on the same unseen future data.
(2) Environmental Settings: All experiments (our PSO–BiLSTM–Attention model and all four baseline models) were executed in the identical hardware and software environment detailed in Section 3.1.2 (Windows 10, Intel Xeon E5-2697 v4, 64 GB RAM, NVIDIA RTX 4090 GPU, Python 3.8, PyTorch 1.12 framework). This eliminates computational environment, random seed initialization, and numerical precision as potential confounding variables.
(3) Hyperparameter Tuning: The comparison is not an “optimized vs. unoptimized” test. We performed systematic hyperparameter tuning for all baseline models to ensure they achieved their best possible performance on our validation set. For ARIMA and SVR, a standard grid search was used. For the deep learning baselines (CNN-LSTM, CNN–BiLSTM), we conducted a robust grid search over key parameters (e.g., learning rate, hidden units, filter sizes).
(4) Model Selection Rationale: Our choice of these four specific baselines is principled and representative. ARIMA: The gold-standard classical time-series model widely used in epidemiological forecasting, providing a traditional statistical benchmark. SVR: A representative traditional machine learning method with proven efficacy in small-sample medical scenarios, capturing nonlinear patterns without deep architectures. CNN-LSTM: A widely adopted hybrid deep learning architecture combining spatial feature extraction (CNN) with temporal modeling (LSTM). CNN–BiLSTM: An advanced bidirectional variant that shares architectural similarities with our model, providing a strong deep learning upper baseline.

This rigorous setup ensures that Table 3 represents a true “apples-to-apples” comparison, and the superior performance of our model is attributable to its architecture and optimization strategy, not to an unfair baseline. Table 3 details the performance of each model across five evaluation metrics.

The comparison results demonstrate that the PSO–BiLSTM–Attention model achieved optimal performance across all evaluation metrics. Compared to the classic ARIMA model, the proposed model reduced MAE from 0.2718 to 0.0164, which is a decrease of 93.96%; MAPE decreased from 79% to 15%, which is a reduction of 81.01%; and R^2^ improved from 0.79 to 0.97, which is an increase of 22.78%. This significant difference indicates that deep learning models possess clear advantages in handling complex nonlinear time series problems, capable of capturing complex patterns that ARIMA models struggle to model. Compared to the traditional machine learning model SVR, the proposed model reduced MAE by 91.44%, MAPE by 75.41%, and improved R^2^ by 10.23%, demonstrating that deep neural networks significantly outperform traditional methods in feature extraction and pattern learning capabilities. In the comparison within deep learning models, the PSO–BiLSTM–Attention model, compared to the CNN–LSTM model, reduced MAE by 86.20%, MSE by 43.20%, MAPE by 44.44%, and improved R^2^ by 4.30%. Compared to the more advanced CNN–BiLSTM model, the proposed model still reduced MAE by 75.99%, MSE by 14.97%, MAPE by 28.57%, and improved R^2^ by 2.11%. These improvements are mainly attributed to three innovative aspects: First, the multi-scale attention mechanism, compared to a single CNN feature extractor, can more flexibly capture feature patterns at different temporal scales. Second, the PSO intelligent optimization algorithm ensures global optimality of model hyperparameter configuration, avoiding the limitations of manual parameter tuning. Finally, the introduction of group-specific layers enables refined modeling for different populations, enhancing the model’s personalized prediction capability.

In summary, both ablation experiments and comparative analysis jointly validate the superior performance of the PSO–BiLSTM–Attention model. The model not only significantly outperforms traditional methods and previous deep learning models across all evaluation metrics but also achieves high-precision prediction for different sex and age groups, demonstrating excellent generalization capability and practical value.

### 3.3. SHAP-Based Model Interpretability Analysis

While the performance metrics in Section 3.2 demonstrate what our model can achieve, this section focuses on how it achieves it. To move beyond a “black box” prediction, we employ the SHAP framework, chosen for its strong theoretical guarantees of consistency and fair attribution based on game theory.

For a systematic analysis, our interpretation is structured into a three-level “global–local–component” framework. This approach allows for a multi-faceted examination of the model, where each subsequent section answers a different question:

(1) Section 3.3.1 (Global Feature Importance): Answers the question, “Overall, which sex-age groups are the most important drivers for the model’s predictions?”
(2) Section 3.3.2 (Local Interpretation): Answers the question, “For a specific high-risk or low-risk prediction, which features pushed the model to that exact decision?”
(3) Section 3.3.3 (Component Contribution): Answers the question, “Internally, how much of the final prediction comes from the simple linear (residual connection) path versus the complex deep learning (BiLSTM–Attention) path?”

This systematic approach allows us to translate the abstract SHAP values into clear, actionable epidemiological and clinical insights”.

#### 3.3.1. Global Feature Importance Interpretation Analysis

**Figure 7:**
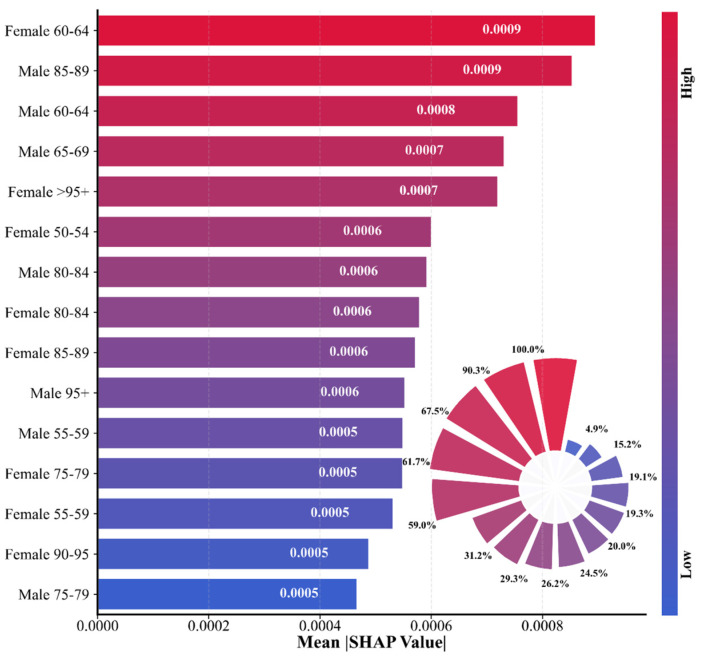
Global feature importance analysis of PSO–BiLSTM–Attention model.

Global feature importance analysis reveals overall contributions of sex-age features in model prediction. Figure 7 (mean SHAP value ranking) displays mean SHAP value rankings for 24 input features. Females aged 60–64 years showed highest importance, followed by males aged 85–89 years and males aged 60–64 years. All top features originate from populations over 60 years, indicating that this age range represents a critical period for IHD prediction. The pie chart shows that females aged 60–64 contribute 10% of total importance, males aged 85–89 account for 9.5%, and combined they represent approximately 20%, highlighting the core predictive value of specific high-risk populations.

**Figure 8:**
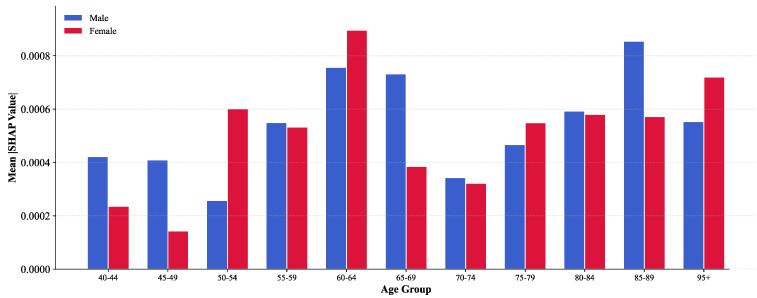
Feature importance by age group and gender.

Figure 8 (stratified importance by age/sex) compares feature importance stratified by age and sex. The 60–64 years age group demonstrates the highest importance in both populations (total 0.001653), significantly exceeding other ranges. This aligns with epidemiological findings: 60–64 years represents a critical turning point for rapid IHD incidence increase, as this group faces physiological decline and substantial social pressure, making them a key prevention target. The 85–89 year group ranks second (0.001426), reflecting elderly population complexity and disease risk heterogeneity. Notably, the 40–49 year group exhibits relatively low importance, suggesting that model predictions for younger populations rely more on long-term trends rather than short-term fluctuations.

Female features demonstrate higher importance in most age groups. In the 60–64 year group, female importance (0.000896) significantly exceeds males (0.000757) by 18.4%, likely related to post-menopausal estrogen decline and weakened cardiovascular protection. Conversely, in the ultra-elderly 85–89 year group, male importance (0.000854) surpasses females (0.000572), indicating more complex disease risk patterns in extremely aged males. This sex–age interaction effect provides data support for formulating differentiated prevention strategies tailored to specific demographic subgroups.

**Figure 9:**
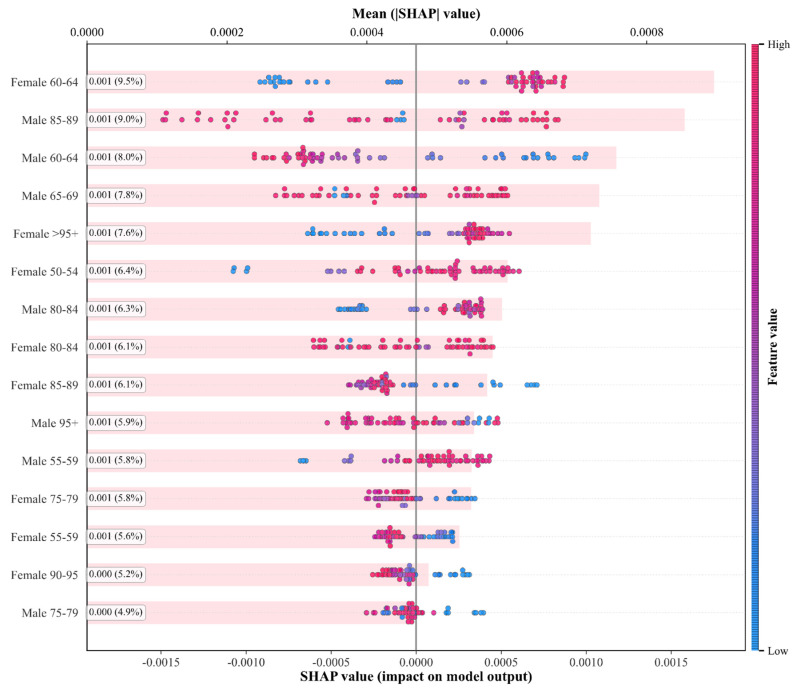
Feature impact distribution.

Figure 9 (feature impact distribution, or beeswarm plot) displays feature influence distribution through a beeswarm plot. The horizontal axis represents SHAP values, the vertical axis shows features ranked by importance, and each scatter point represents a sample. Color coding indicates feature magnitude: deep red for high values; deep blue for low values. For the female 60–64 year group, SHAP values concentrate in the positive region, with high feature values (red points) corresponding to larger positive SHAP values, indicating increased incidence rates significantly elevate prediction output. Conversely, younger age group features exhibit negative distributions, suggesting that increases in these features reduce overall predicted risk. The beeswarm plot reveals nonlinear relationships, with some features showing large SHAP value variance in moderate ranges, reflecting complex model-captured interactions.

Epidemiologically, the 60–64 year group’s high importance relates to combined physiological, psychological, and social factors. Physiologically, atherosclerosis accelerates and vascular elasticity decreases; psychologically, occupational stress and family burdens trigger stress responses; socially, unhealthy lifestyle effects manifest concentratedly. The model automatically identifies this age group as a key predictive factor, providing clear targets for clinical screening and early intervention.

#### 3.3.2. Local Interpretation Analysis of Prediction Samples

**Figure 10:**
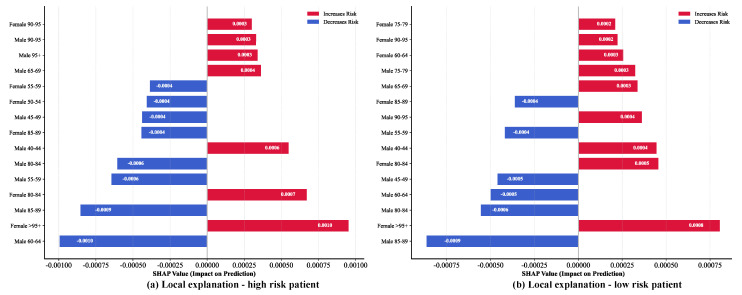
Local interpretation of model predictions.

Local interpretation analysis focuses on individual prediction samples, revealing how the model makes decisions based on specific feature combinations. Figure 10 displays SHAP value decomposition for two typical cases: a high-risk patient (left panel) and a low-risk patient (right panel).

For the high-risk patient, predictions are primarily driven by ultra-elderly features: females aged 95+ (SHAP = +0.0010), females aged 90–95 (+0.0003), males aged 95+ (+0.0003), and males aged 65–69 (+0.0004) exert positive effects, indicating that elevated incidence rates in ultra-elderly populations drive high-risk classification. Conversely, females aged 60–64 (SHAP = −0.0010) and males aged 85–89 (−0.0009) show negative contributions, providing offsetting effects. This complex positive–negative interaction reflects cross-age and cross-sex association patterns learned by the model. For the low-risk patient, the pattern reverses. Multiple elderly male features—males aged 85–89 (−0.0009), 60–64 (−0.0005), and 80–84 (−0.0006)—demonstrate negative contributions. Although ultra-elderly features generate positive effects, they are offset by multiple negative features.

It is important to clarify an apparent contradiction between the global and local plots. The beeswarm plot (Figure 9) shows that the “Female 60–64” feature has a strong average positive SHAP value (high feature values push the prediction higher). However, in the local plot (Figure 10a), this same feature can contribute negatively. This is not a contradiction; it is the essence of SHAP’s ability to capture interaction effects. The global plot shows the feature’s main effect in isolation. The local plot shows its effect in the context of one specific sample. In Figure 10a, the “Female 60–64” feature interacts with other, highly dominant features (e.g., “Female 95+”). The model has learned a complex rule that when all elderly groups are high, the relative contribution of the F_60–64 group is diminished or even reversed to provide a balancing effect, preventing an unrealistically high prediction. This demonstrates the model’s ability to learn nuanced, nonlinear feature interactions.

Local interpretation provides evidence for personalized risk assessment, enabling clinicians to develop targeted interventions. For high-risk predictions, strengthen chronic disease management for ultra-elderly populations; for low-risk predictions, adjust resource allocation toward other high-risk groups.

#### 3.3.3. Analysis of Architectural Component Contribution 

**Figure 11:**
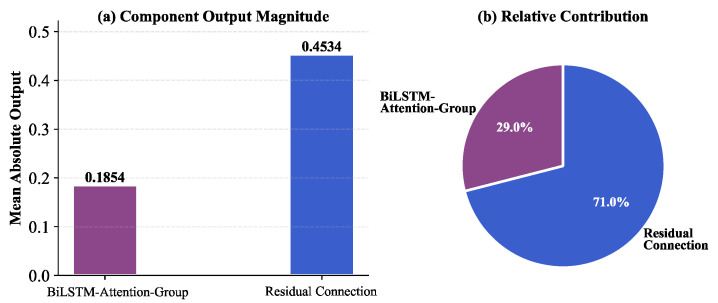
Architectural component contribution analysis.

This study analyzes contributions of different architectural components to understand the PSO–BiLSTM–Attention model’s internal mechanisms. Figure 11 quantitatively compares the BiLSTM–Attention–Group main pathway and residual connection pathway.

To quantify these contributions, we analyzed the two terms from Equation (20) separately. For every prediction in the test set, we saved the output of the BiLSTM–Attention–Group component and the output of the residual connection component before they were fused (Equation (20)). Figure 11 plots the mean absolute value of these two components across all test samples.

Quantitative analysis reveals that the residual connection component generates a mean absolute output of 0.4534 (71.0% contribution), while BiLSTM–Attention–Group produces 0.1854 (29.0%). This highlights the dual-pathway strategy: the residual connection pathway, which is a linear projection of the averaged input features (Equation (20)), is expertly designed to capture the primary, stable trends and baseline autocorrelation in the time series. This is consistent with epidemiological principles where long-term trends are stable. By “offloading” this primary trend to the residual path, the model frees the deep BiLSTM–Attention–Group pathway (29.0% contribution) to focus on what it does best: modeling the complex, nonlinear temporal dependencies, local fluctuations, and cross-group interactions that the linear path cannot capture. This dual-pathway fusion achieves an optimal “stability + precision” balance, which is the core mechanism enabling its excellent performance.

## 4. Conclusions

Based on the GBD 2021 time series data from 1990 to 2021, this study constructed a PSO–BiLSTM–Attention hybrid deep learning model with SHAP interpretability analysis, achieving accurate prediction and transparent explanation of IHD incidence rates across 24 sex-age stratified groups. The model achieves MAE of 0.0164 and R^2^ of 0.97, significantly outperforming traditional ARIMA models (93.96% MAE reduction) and previous CNN–BiLSTM approaches (75.99% MAE improvement). SHAP analysis reveals that females aged 60–64 years and males aged 85–89 years are core predictive factors, accounting for nearly 20% of total feature importance, consistent with epidemiological patterns of post-menopausal cardiovascular risk acceleration and ultra-elderly multimorbidity complexity. Architectural analysis demonstrates that residual connections capture 71% of contribution through major trend modeling, while BiLSTM–Attention pathways extract 29% through complex nonlinear pattern learning, achieving optimal stability–precision balance.

This research provides important guidance for IHD prevention and control. At the policy level, identified high-risk populations provide quantitative basis for resource allocation prioritization and precise medical deployment. Clinically, fine-grained predictions support screening protocols, early intervention timing, and individualized treatment planning, with local SHAP enabling patient education about modifiable risk factors and promoting treatment adherence. Methodologically, the successful integration of intelligent optimization, deep learning, and transparent explanation validates the effectiveness of this technical approach, providing a paradigm for chronic disease prediction research and advancing responsible AI application in public health.

However, this study has several important limitations. First, we must address the “ecological fallacy”: our model uses aggregated, population-level GBD data (ASIRs), not clinical risk factors (e.g., blood pressure, cholesterol). Therefore, its findings apply to population-level risk trends, not the diagnosis or screening of individuals. The model identifies which demographic groups are at highest risk, not which individuals within those groups are. Second, our model, like all deep learning approaches, is data-hungry and its performance relies on long-term, high-quality time series. Third, while SHAP provides strong interpretability, it is an approximation method and its computational cost for complex models is high. Finally, the PSO algorithm, while effective, does not guarantee finding the absolute global optimum. To address these limitations and extend this work, future research should integrate individual-level multimodal data (e.g., EHR, genomics) for precision prediction, incorporate causal inference methods for policy evaluation, develop real-time early warning systems, and create interactive decision-support tools. These advances will enhance predictive capability, interpretability depth, and practical value, ultimately serving cardiovascular disease prevention objectives and health strategy implementation.

## Figures and Tables

**Table 1:** Justification of key methodological and model parameters.

Parameter/Method	Selected Value	Justification and Rationale
Sliding Window	10 years	Balances the need for sufficient historical context (long-term trends) with sensitivity to more recent patterns. Shorter windows (e.g., 3–5 years) were found to be too volatile, while longer windows smoothed out important recent dynamics.
Dataset Split	8:2 Sequential (1990–2016 Train, 2017–2021 Test)	This “walk-forward” split is standard for time series forecasting. It strictly prevents information leakage (using future data to predict the past) and tests the model on the most recent, unseen data.
PSO Population	Eight Particles	A small population size was chosen to balance exploration of the search space with the high computational cost of training a deep learning model for each particle in each iteration.
PSO Iterations	20 Iterations	The fitness (validation RMSE) was observed to converge and stabilize by the 20th iteration, indicating further searching yielded diminishing returns.
Evaluation Criteria	MAE, MSE, RMSE, MAPE, R^2^	Using this comprehensive suite of five standard metrics provides a holistic view of the performance, capturing accuracy (MAE, RMSE), relative error (MAPE), and goodness-of-fit (R^2^), rather than relying on a single, potentially misleading, metric.

**Table 2:** Ablation experiments: comparison of baseline model prediction performance.

Model	MAE	MSE	RMSE	MAPE	R^2^
LSTM	0.1106	0.1321	0.1542	0.37	0.86
BiLSTM	0.0994	0.1005	0.1397	0.35	0.89
BiLSTM–Attention	0.0919	0.0769	0.1039	0.32	0.92
PSO–BiLSTM	0.0678	0.0561	0.0782	0.27	0.94
PSO–BiLSTM–Attention	0.0164	0.0426	0.0206	0.15	0.97

**Table 3:** Comparison of prediction performance with previous research models.

Model	MAE	MSE	RMSE	MAPE	R^2^
ARIMA	0.2718	0.1722	0.2177	0.79	0.79
SVR	0.1915	0.1114	0.1387	0.61	0.88
CNN–LSTM	0.1188	0.0750	0.0745	0.27	0.93
CNN–BiLSTM	0.0683	0.0501	0.0504	0.21	0.95
PSO–BiLSTM–Attention	0.0164	0.0426	0.0206	0.15	0.97

## Data Availability

For inquiries related to the availability of the original data, please contact the corresponding author. The data are not publicly available due to restrictions on the redistribution of aggregated data mandated by the GBD Study data access policy.

## Abbreviations

ADF	Augmented Dickey–Fuller
ARIMA	Autoregressive Integrated Moving Average
ASIR	Age-Standardized Incidence Rate
BiLSTM	Bidirectional Long Short-Term Memory
CNN	Convolutional Neural Network
CVD	Cardiovascular Diseases
EWMA	Exponentially Weighted Moving Average
GBD	Global Burden of Disease
GHDx	Global Health Data Exchange
GM	Gray Model
IHD	Ischemic Heart Disease
IQR	Interquartile Range
LSTM	Long Short-Term Memory
MAE	Mean Absolute Error
MAPE	Mean Absolute Percentage Error
MSE	Mean Squared Error
PSO	Particle Swarm Optimization
R^2^	Coefficient of Determination
ReLU	Rectified Linear Unit
ResNet	Residual Network
RMSE	Root Mean Squared Error
RNN	Recurrent Neural Network
SHAP	SHapley Additive exPlanations
SVR	Support Vector Regression
WHO	World Health Organization
XGBoost	Extreme Gradient Boosting
